# Expansion of Lymphocytes from Prostatic Adenocarcinoma and Adjacent Nonmalignant Tissue

**DOI:** 10.1155/2022/6499344

**Published:** 2022-06-16

**Authors:** Linh T. Nguyen, Charlotte S. Lo, Michael Fyrsta, Jessica Nie, Jennifer Y. Yam, Pei-Hua Yen, Michael X. Le, Karen Hersey, Miran Kenk, Megan Crumbaker, Neil Fleshner, Girish Kulkarni, Robert Hamilton, Michael Jewett, Antonio Finelli, Andrew Evans, Joan Sweet, Pamela S. Ohashi, Anthony M. Joshua

**Affiliations:** ^1^Princess Margaret Cancer Centre, University Health Network, Toronto, Canada; ^2^Kinghorn Cancer Centre, St Vincent's Hospital, Garvan Institute of Medical Research, Sydney, Australia; ^3^Mackenzie Health, Department of Pathology, Cortellucci Vaughan Hospital, Vaughan, Canada; ^4^Department of Immunology, University of Toronto, Toronto, Canada

## Abstract

**Background:**

The evaluation of tumour-infiltrating lymphocytes (TILs) in solid malignancies has yielded insights into immune regulation within the tumour microenvironment and has also led to the development and optimisation of adoptive T cell therapies.

**Objectives:**

This study examined the *in vitro* expansion of TILs from prostate adenocarcinoma, as a preliminary step to evaluate the potential of TILs for adoptive T cell therapy. *Design, Setting, and Participants*. Malignant and adjacent nonmalignant tissues were obtained from fifteen men undergoing radical prostatectomy. *Interventions*. There were no study interventions. *Outcome Measurements and Statistical Analysis*. Expanded cells were analysed by flow cytometry, and the data was assessed for associations between cell subpopulations and expansion rate.

**Results:**

Tumour-infiltrating lymphocytes could be expanded to numbers that would be needed to generate a therapeutic infusion product from nine of 15 malignant specimens (60%). The CD4^+^ T cells predominated over CD8^+^ T cells (median 56.8% CD4^+^, 30.0% CD8^+^), and furthermore, faster TIL expansion was associated with a higher proportion of CD4^+^ T cells (median 69.8% in faster-growing cultures; 36.8% in slower-growing cultures). A higher proportion of CD3^−^CD56^+^ cells versus CD3^+^ cells was associated with slower TIL expansion in cultures from malignant specimens (median 13.3% in slower-growing cultures versus 2.05% in faster-growing cultures), but not from nonmalignant specimens.

**Conclusions:**

The expansion of TILs for potential therapeutic use is feasible. Our findings also indicate that further examination of TILs from prostate adenocarcinomas may yield insights into mechanisms of regulation of T cells within the tumour microenvironment. Further research is required to evaluate their therapeutic potential.

## 1. Introduction

Adoptive T cell therapy is an active area of investigation for the treatment of solid cancers [1, 2]. Early success in the field was demonstrated using tumour-infiltrating lymphocytes (TILs), where the T cell infiltrate from tumour specimens is activated and expanded *in vitro* and infused in an autologous manner following lymphodepleting chemotherapy [3, 4]. The durable clinical responses reported using this approach has led to clinical studies of TIL-based therapy for several malignancies [5–10]. Other approaches of adoptive T cell therapy include genetic engineering of T cells to express a chimeric antigen receptor (CAR) or a T cell receptor (TCR) that enables recognition of cancer cells by the T cells [11]. These approaches have also shown clinical efficacy with several CAR-T cell products now approved as standard-of-care. One advantage of TIL-based adoptive T cell therapy is that the TCR repertoire in the cell infusion product is polyclonal and is derived from the patient's endogenous repertoire. These features enable the targeting of multiple tumour-associated antigens thereby decreasing immune escape by cancer cells, with a lower risk of on-target off-tumour toxicities that can be seen with engineered receptors. However, TIL-based therapy requires that the cancer type harbours TILs that can be ready expanded *in vitro*.

Prostate cancer was the first malignancy to demonstrate improved survival with a cancer-specific vaccine (Sipuleucel-T) [12]. However, little progress has been made since with immunotherapeutics [13–16]. Two previous studies that evaluated the *in vitro* expansion of TILs from prostate tumours indicated that TILs have the potential for adoptive T cell therapy for prostate cancer [17, 18]. In one study by Haas et al., tumour samples from four patients with prostate cancer were evaluated: different types of culture media were compared, and culture times ranged from 51 to 77 days. In the second study by Yunger et al., tumour samples from eight patients were evaluated using contemporary culture methods. After plating 18–34 tissue fragments from each sample, at least three fragments from each tumour sample showed TIL outgrowth within 2–4 weeks, with CD4^+^ T cells generally being favoured over CD8^+^ T cells. In this current study, we expand on the findings from Haas et al. and Yunger et al. in a larger number of samples and explore further associations.

We assessed the ability of TILs to expand from tissue specimens obtained from 15 men undergoing a radical prostatectomy (RP) for prostate adenocarcinoma, including a subset treated with neoadjuvant androgen-deprivation therapy (ADT) [19–21]. Given the evidence for a field effect of cancerisation in prostate cancer [22], we also assessed the expansion of T cells from adjacent nonmalignant regions from 13 of the resections.

## 2. Material, Patients, and Methods

### 2.1. Patients and Tissue Samples

This study was approved by the Research Ethics Board (REB) of the University Health Network (UHN). All patients provided written informed consent for the collection of samples and subsequent analysis. Patients underwent open RP at UHN between May 2012 and October 2013. Neoadjuvant ADT was administered to five of the patients under an open label study (NCT01674279). Frozen sections were evaluated immediately following surgical resection to identify malignant and nonmalignant areas, followed by sampling for TIL expansion. Tissues were immediately transported in sterile saline for initiation of TIL cultures from fresh samples.

### 2.2. Media

Complete Medium (CM) for T cell culturing consisted of Iscove's modified Dulbecco's medium (IMDM) (Lonza, cat# 12440-079) with 10% human serum or plasma (prepared in-house from healthy donors), 25 mM HEPES (Gibco, cat# 17-737E), 100 U/ml penicillin/100 *μ*g/ml streptomycin (Lonza, cat# 17-602E), 10 *μ*g/ml gentamicin sulphate (Lonza, cat# 17-519L), 2 mM L-glutamine (Lonza, cat# 17-605E), 5.5 × 10−5 M 2-mercaptoethanol (Invitrogen, cat# 21985-023), and 6000 IU/ml human recombinant interleukin-2 (IL-2) (Novartis).

### 2.3. T Cell Culturing

One 1 mm^3^ fragment of tumour tissue was placed per well in 24-well plates. Cells were cultured with 2 ml of CM per well in a 37°C, 5%CO_2_, humidified incubator. After the first week in culture, 1 ml of CM from each well was replaced with fresh CM three times a week, and once cells began to proliferate, wells were pooled prior to expansion and replated at a concentration of approximately 0.5 × 10^6^ cells/ml. Cultures were generally expanded for four weeks, until either a maximum of approximately 1.5 × 10 [8] cells were obtained, or until the expansion rate slowed down. Cells were analysed on the day of harvest by flow cytometry, and the remaining cells were cryopreserved in 10%DMSO/90% human AB serum (OriGen, cat# CP-50, GeminiBio 100–512, respectively).

### 2.4. Flow Cytometry

Cells were stained at 4°C for 30 minutes with the selected antibodies in phosphate buffered saline (PBS, Gibco cat# 10010-023) containing 2% fetal calf serum (Gibco, cat# 12483020) and 0.05% sodium azide (Sigma, cat# S2002). Cells were then washed and resuspended in 1% paraformaldehyde + PBS for acquisition (ThermoFisher, cat# J19943-K2). Antibodies used included CD3-phycoerythrin (PE) (BD, cat# 555333), CD4-fluorescein isothiocyanate (FITC) (BD, cat# 555346), CD8-peridinin chlorophyll protein (PerCP) (BioLegend, cat# 301030, CD56-allophycocyanin (APC) (BD, cat# 555518), CD19-FITC (BD, cat# 555412), and CD14-PerCP-Cyanine5.5 (BD, cat# 550787). Data was acquired on a FACSCalibur flow cytometer (BD) and analysed using FlowJo software.

### 2.5. Rapid Expansion Protocol

Cryopreserved TILs were thawed. REPs were initiated in media consisting of Complete Media: AIM V media (Gibco, cat# 12055091) at a 50 : 50 ratio, 30 ng/ml OKT3 (Miltenyi Biotec, cat# 170-076-124), 600 IU/ml IL-2 (Novartis), and allogeneic PBMCs (irradiated 50 Gy) at a ratio of 1 TIL : 200 feeder cells. 600 IU/ml IL-2 was used instead of 6000 IU/mL based on in-house data that showed similar expansion with both concentrations (unpublished). On day 5, 80–90% of media were replaced with fresh media (50 : 50 CM : AIM V with 600 IU/ml IL-2), and on day 7 onwards, cells were expanded using AIM V with 5% human serum with 600 IU/ml IL-2.

## 3. Results

Malignant tissues were collected from radical prostatectomy (RP) from 15 patients. For 13 of the specimens, adjacent nonmalignant tissues were also analysed. Five of 15 patients had received six months of neoadjuvant androgen-deprivation therapy (ADT) as part of an open label study (NCT01674279). Clinicopathological features for the 15 cases are summarised in Table 1.

**Figure 1:**
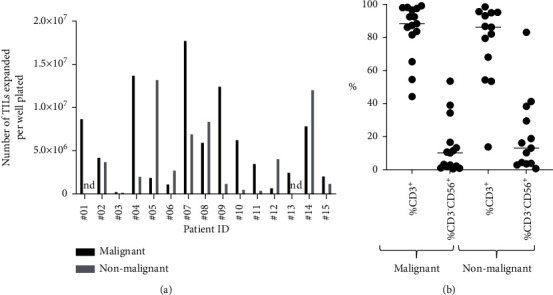
Properties of TIL cultures. (a) The number of TILs expanded from malignant and adjacent nonmalignant tissues is shown. Numbers of TILs are normalized to one starting well for each tissue sample. nd, not done. (b) The proportion of CD3^+^ T cells and CD3^−^CD56^+^ cells were quantified by flow cytometry at the time of TIL culture harvest.

T cells were expanded from tissue samples using the T cell growth factor interleukin-2 (IL-2) as previously described [23]. T cell cultures were initiated by plating one tissue fragment per well. The number of wells at the start of culture was on average 10.6 (SD 5.8; range 4–24). The average number of days in culture was 27 (SD 8.0, range 12–42). At the end of the culture period, cells were harvested and analysed for surface markers (CD3, CD4, CD8, CD56, CD19, and CD14). Data from T cell culturing are shown in Table 2. The expansion rate of T cells was variable amongst samples. Some cultures exhibited almost no expansion, while others reached over 1 × 10 [8] cells within a few weeks. This variability was not due to the variation in the number of wells seeded, since a similar pattern of expansion is observed if the cell count data is normalized to one initial well across all samples (Figure 1(a)). T cell expansion was also robust from some of the nonmalignant tissue samples.

To assess the cellular composition of the expanded T cell cultures, cells were stained and analysed by flow cytometric analysis. As expected, most TIL cultures were composed predominantly of CD3^+^ T cells (Figure 1(b)). A minority of cells were CD3^−^ CD56^+^ cells which are also known to proliferate in the presence of IL-2. The cultures were also stained for CD19 and CD14 to enumerate B cells and monocytes, respectively. As expected, all cultures had negligible (<2%) staining of the B cell marker CD19^+^ and the monocyte marker CD14^+^, except for two of the cultures which had low expression: #11-Malignant was 2.3% CD19^+^ and 4.2% CD14^+^, and #15-Malignant was 2% CD19^+^ and 2% CD14^+^. The percentage of CD3^+^ cells was also examined in cultures from the matched adjacent nonmalignant tissue samples (Figure 1(b)). These cultures were also predominantly CD3^+^, with levels similar to the matched tumour-derived cultures with a few exceptions, mostly notably the culture from patient #09 which was 83% CD3^−^CD56^+^ cells.

**Figure 2:**
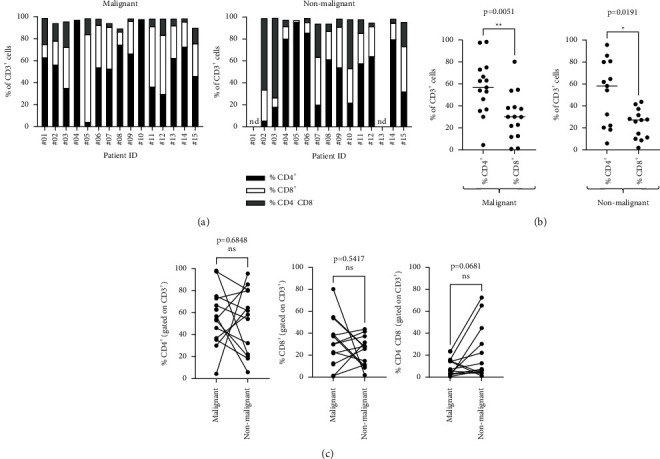
A prominent population of CD4^+^ T cells are found in TILs from prostate tissues. (a) The proportion of CD4^+^, CD8^+^, and CD4^−^CD8^−^ cells in the CD3^+^ population is shown for the TIL culture expanded from each tissue specimen. nd, not done. (b) The proportion of CD4^+^ T cells was compared to CD8^+^ T cells in TIL cultures from malignant and nonmalignant tissues (Mann-Whitney, two-tailed). (c) The proportions of CD4^+^ T cells, CD8^+^ T cells, and CD4^−^CD8^−^ T cells were compared between malignant tissues and matched nonmalignant tissues (paired *t*-test, two-tailed).

The proportions of CD8^+^ putative cytotoxic T cells and CD4^+^ putative helper T cells in cell cultures were compared. When examining the proportion of CD4^+^ and CD8^+^ T cells, cultures generally consisted of a mix of CD4^+^ and CD8^+^ T cells, with variable ratios across cultures from different patients (Figure 2(a)). However, overall, the proportion of CD4^+^ T cells was higher than the proportion of CD8^+^ T cells. This was the case in cultures from malignant tissues (median 56.8% CD4^+^, 30.0% CD8^+^, *p*=0.0051) as well as nonmalignant tissues (median 58.2% CD4^+^, 27.20% CD8^+^*p*=0.0191) (Figure 2(b)).

When examining cultures from malignant tissues to cultures from nonmalignant tissues, the proportion of CD4^+^ and CD8^+^ T cells did not differ (Figure 2(c)). Notably, some cultures had a population of CD3^+^ T cells that did not express CD4 or CD8 coreceptors (double negative T cells). There tended to be a higher proportion of these atypical CD4^−^CD8^−^ double negative T cells in cultures from the nonmalignant tissues compared to the matched malignant tissues; however, the difference did not reach statistical significance (*p*=0.0681) (Figure 2(c)).

**Figure 3:**
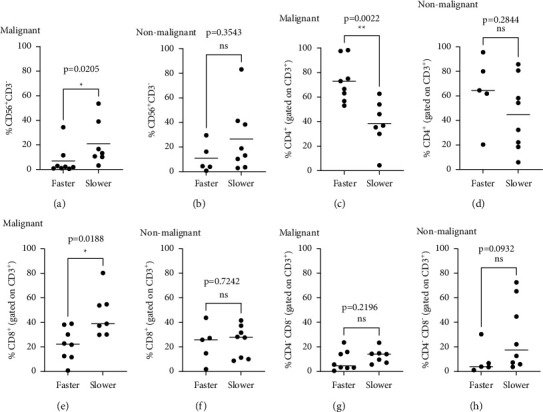
CD3^−^ CD56^+^ cells are associated with reduced TIL growth, whereas CD4^+^ T cells are preferentially found in faster-growing cultures. ILC (CD3^−^CD56^+^) (a, b), CD4^+^ (c, d), CD8^+^ (e, f), and CD4^−^CD8^−^ (g, h) subsets were quantified in TIL cultures according to their rates of expansion (faster or slower). Analysis was performed for malignant and nonmalignant TIL cultures as indicated. Two-tailed Mann-Whitney tests were used for A-H.

We have previously reported on a population of regulatory innate lymphoid cells (ILCreg) (CD3^−^ CD56^+^ cells) identified in ovarian cancer TIL cultures [24], where ILCregs dampen the expansion of T cells. Faster-growing cultures from prostatic tissue had a median of 2.05% CD3^−^ CD56^+^ cells (range 0.5%–34.4%), whereas slower-growing cultures had a median of 13.3% CD3^−^ CD56^+^ cells (range 3.2%–53.6%); slower expansion was defined as cultures that reached less than 4 × 10 [6] cells per initial well seeded. Thus, a higher proportion of CD3^−^ CD56^+^ cells was present in the prostate TIL cultures that exhibited slower expansion (*p*=0.0205) (Figure 3(a)). This observation in prostate TILs is consistent with our report in TILs from ovarian cancer [24]. In contrast, when the same analysis was done for CD3^−^CD56^+^ cells in cultures from adjacent nonmalignant tissue, we did not see the same association that we saw in cultures from malignant tissues (Figure 3(b)). Therefore, CD3^−^CD56+ cells corresponded with reduced T cell proliferation, and notably this association was only found within the tumour.

CD4^+^, CD8^+^, and CD4^−^CD8^−^ T cell populations were also analysed for any association with slower or faster growth rates. In TIL cultures from malignant tissues, faster expansion (defined by reaching between 4 × 10 [6] and 3 × 10 [7] cells per initial well seeded) was associated with a higher proportion of CD4^+^ T cells (*p*=0.0022) (Figure 3(c)): the median percentage of CD4^+^ cells in faster-growing cultures was 69.8%, versus 36.8% in slower-growing cultures (range 53.0%–98.3% and 4.4%–62.7%, respectively). Correspondingly, faster-growing cultures had a lower proportion of CD8^+^ T cells (*p*=0.0188) (Figure 3(e)). This observation was not apparent in T cell cultures from nonmalignant tissues, where neither CD4^+^ nor CD8^+^ T cells were associated with growth rate (Figures 3(d) and 3(f)). When evaluating CD4^−^CD8^−^ double negative T cells, no association was found with growth rate, whether in malignant specimens or nonmalignant specimens (Figures 3(g) and 3(h)).

Of the 15 patients included in this study, five were treated with neoadjuvant ADT. Comparison of the TIL cultures from these two small cohorts (ADT versus no ADT) showed that they did not differ in any of the following features: TIL expansion rate (*p*=0.3097) and proportion of CD4^+^ T cells (*p*=0.3097), CD8^+^ T cells (*p*=0.3097), and CD4^−^CD8^−^ T cells (*p*=0.6787) (data not shown). Analysis of a larger cohort would be required to further explore whether ADT impacts TIL expansion or phenotype.

**Figure 4:**
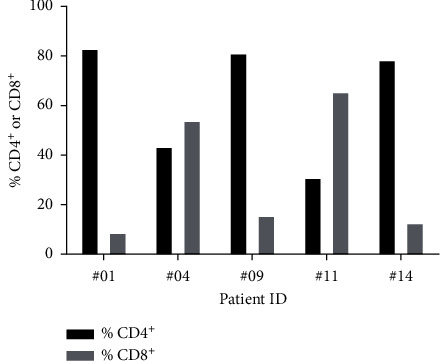
T cell populations after a TIL rapid expansion protocol (REP). CD4^+^ and CD8^+^ TILs were analysed by flow cytometry after 14 days in a REP.

Current protocols for generating TILs for therapeutic infusion require TILs to be expanded using a “Rapid Expansion Protocol” (REP) after the initial expansion of TILs using IL-2. The ability of prostate TILs to expand in a REP was evaluated using TILs from five patients. After stimulation with anti-CD3 antibody in the presence of IL-2 and irradiated feeder cells (allogeneic peripheral blood mononuclear cells), the TILs expanded a median of 720-fold over 14 days of the REP (range 130- to 1193-fold) (data not shown). At the end of the REP, the cells were almost all CD3+ T cells, as expected (mean 98.0% ± 1.0% SD), with a variety of CD4^+^ and CD8^+^ T cell populations (Figure 4).

## 4. Discussion

This study evaluated the *in vitro* expansion of TILs from prostatic adenocarcinoma. It was found that TILs could be expanded from almost all specimens, and in some cases large numbers of cells were generated within four weeks. The expanded cells were predominantly CD3^+^ T cells. The proportions of CD4^+^ and CD8^+^ T cell subpopulations were variable, but there was a higher proportion of CD4^+^ T cells overall, compared to CD8^+^ T cells. Furthermore, TIL cultures that exhibited faster expansion rates were more CD4-dominant than those with slower rates of expansion. Finally, the association of CD3^−^CD56^+^ cells with slower TIL expansion suggests that ILCs may play a negative regulatory role in TIL expansion in prostatic adenocarcinoma.

CD8^+^ T cells are conventionally considered the main mediators of antitumour immunity, and there is some evidence that clinical responses after therapeutic TIL infusion are associated with a higher proportion of CD8^+^ T cells infused (versus CD4^+^ T cells) [25]. However, other studies show that CD4^+^ T cells can also be effective [5, 26]. Data from expansion of “pre-REP” TILs from 11 TIL cultures established from two prostatic adenocarcinoma patients reported by Yunger et al. showed that those two cultures were CD4-skewed [18]. Haas et al. expanded TILs from four prostate carcinoma specimens [17]. In that study, histological staining of cryopreserved tissues showed that CD4^+^ T cells were the predominant cell population in prostate carcinomas. The data from our study shows that the proportion of CD4^+^ versus CD8^+^ T cells in TIL cultures is variable. It is expected that there is interpatient and intratumoural heterogeneity in T cell subsets *in situ* and in their *in vitro* proliferation. The fact that the proportion of CD4^+^ T cells was of statistical significance led us to investigate whether this could have any biological impact. As shown in Figure 3(c), in malignant specimens, a higher proportion of CD4^+^ T cells was associated with a faster TIL expansion rate. To our knowledge, the association of faster TIL expansion rate with CD4-skewed TIL cultures has not previously been reported. We have also observed this phenotype in breast cancer (Warner and Ohashi, not published). Studies are underway to investigate the mechanism and implication of this observation.

The design of this study allowed for a comparison of adjacent histologically nonmalignant tissue with matched malignant tissue from the same surgical procedure. Despite the fact that there might be “premalignant” changes present in these nonmalignant tissues, nevertheless, our analysis revealed some differences. For example, our data indicates that T cells from nonmalignant tissues are more likely to be enriched with atypical CD4^−^ CD8^−^ double negative T cells. This enrichment could be due to *in situ* differences in T cell subsets, or differential expansion rates during culture. Double negative T cells have been described in several contexts, ranging from negative regulation of immune responses to mediators of anticancer responses [27]. Future studies are needed to evaluate their mechanism of enrichment and physiological significance in the context of nonmalignant prostate tissue. Another difference between the cultures from malignant tissues and nonmalignant tissues was that only the former showed evidence of regulation of TIL expansion by CD3^−^CD56^+^ cells. We have previously described a regulatory role for CD3^−^CD56^+^ ILCregs in TIL cultures from ovarian cancer samples [24]. We found that this population inhibited expansion and altered the cytokine production of CD4^+^ and CD8^+^ T cells *in vitro*. Importantly, we also demonstrated that the CD3^−^CD56^+^ cells found in the rapidly growing cultures were not able to inhibit proliferation. Other studies describe immunosuppressive properties for CD56^+^ ILC populations found in breast cancer non-small cell lung cancer and sarcoma patients [28–30]. Collectively, these studies suggest that CD56^+^CD3^−^ cells play a critical role in regulating antitumour immunity. Since our study only found evidence for ILCregs in prostatic malignant tissues and not nonmalignant tissues, this suggests that this regulatory mechanism may be unique to a malignant microenvironment, at least for prostate tissues.

Interestingly, in our study, there were no apparent differences in TIL expansion in patients who received preoperative ADT compared with patients who did not. Previous work has suggested that androgen-deprivation therapy may increase inflammatory T cell responses; for example, androgen-deprivation therapy led to an increase in the numbers of circulating naïve T cells and a T-helper1-biased phenotype shortly after beginning androgen-deprivation therapy [21] and decreased numbers of circulating CD4^+^ T regulatory cells [20]. In studies using short-term androgen-deprivation therapy prior to prostatectomy, an increase in oligoclonal T-cell infiltration into prostate tissue was observed [19]. In a retrospective series of 35 patients treated with neoadjuvant ADT prior to prostatectomy and 40 control patients, Gannon et al. previously showed that ADT may increase the inflammatory infiltrate in treated prostate tumours with increased numbers of CD3^+^, CD8^+^, and CD69^+^ cells seen [31]. Our study used a different read-out for T cell responses and had a limited sample size.

Adoptive cell therapy using TILs for various malignancies is currently under investigation. In this study, only a small proportion of excised tissue was used to evaluate TIL expansion, since there was a concern that the amount of tissue used for research purposes might otherwise affect pathology results. Manufacturing protocols to generate TILs for administration often require expansion to a minimum of 5 × 10 [7] TILs after an initial stage of expansion. Nine of 15 malignant specimens reached this threshold, despite the small volume of the malignant tissue being available for TIL expansion (median of approximately 0.12 cubic cm). Thus, our study adds to the results from other studies in which a smaller number of specimens were examined [17, 18]. Other studies have shown that TILs expanded from prostatic adenocarcinoma can recognize autologous tumour [18]; however, the volume of tissues available for our study did not allow for generating autologous tumour cell targets. Future work may include evaluating the antigen specificity of prostate TILs and exploring strategies to augment the infiltrate into malignant tissues [32].

## 5. Conclusions

This study demonstrates that expansion of prostate TILs for use in adoptive T cell therapy is feasible and suggests further avenues of investigation related to the immune microenvironment of prostate adenocarcinoma.

## Figures and Tables

**Table 1:** Clinicopathological features.

Patient ID	Age at surgery	Pathological stage	Margin status	Gleason score^1^	Other pathological features	PSA level (preoperative or pre-ADT)	PSA levels after surgery	PSA recurrence	PSA at last follow-up^2^
*Did not receive neoadjuvant androgen-deprivation therapy:*									
#01^*∗*^	62	T3b	+	7	EPE, SV	55.61	0.14	Yes	1.7
#02	51	T2c	−	6	PNI	7.13	<0.05	No	<0.05
#03	65	T1c	+	7	PNI	6.13	<0.05	Yes	0.037
#04	64	T2c	−	6	PNI	5.25	<0.05	No	<0.05
#05	62	T2	−	7	EPE, PNI	3.16	<0.05	No	<0.05
#06	62	T2	−	7	PNI	6.26	<0.05	No	<0.05
#07	66	T2	−	7	Nil	17.6	<0.04	No	<0.05
#08	69	T3b	−	7	PNI, EPE, SV	24.64	0.27	No	0.24
#09	64	T3a	−	7	PNI, EPE	10.07	<0.05	Yes	0.12
#10	50	T3a	+	7	PNI	45.28	0.056	Yes	<0.05

*Received neoadjuvant androgen-deprivation therapy:*									
#11	59	T2c	−	n/a	NiI	5.38	<0.05	No	<0.05
#12	69	T2c	−	n/a	PNI	13.84	<0.05	Yes	0.14
#13^*∗*^	62	T2c	−	n/a	PNI	10.19	<0.05	No	<0.05
#14	49	T3a	−	n/a	PNI	247.4	0.08	Yes	0.08
#15	67	T3b	+	n/a	PNI, EPE, SV (bilat)	33.88	<0.05	Yes	<0.05

ADT, androgen-deprivation therapy; EPE, extraprostatic extension; PNI, perineural invasion; PSA, prostate-specific antigen; SVI, seminal vesicle involvement. ^*∗*^Only malignant tissue was available; that is, matched nonmalignant tissue was not assessed. ^1^Gleason score not assessed (n/a) in those treated with neoadjuvant androgen-deprivation therapy. ^2^Median time to follow-up after surgery was 887 days.

**Table 2:** T cell cultures.

Patient ID	Type of tissue	Number of wells initiated for T cell culture	Days in culture	Number of cells obtained after culture
#01	Malignant	12	21	1.05*E* + 08
#01	Nonmalignant	n/a	n/a	n/a
#02	Malignant	14	33	5.98*E* + 07
#02	Nonmalignant	14	25	5.30*E* + 07
#03	Malignant	12	42	3.60*E* + 06
#03	Nonmalignant	12	42	3.00*E* + 06
#04	Malignant	8	35	1.10*E* + 08
#04	Nonmalignant	12	32	2.52*E* + 07
#05	Malignant	24	35	4.70*E* + 07
#05	Nonmalignant	12	26	1.60*E* + 08
#06	Malignant	10	33	1.20*E* + 07
#06	Nonmalignant	10	33	2.80*E* + 07
#07	Malignant	4	33	7.10*E* + 07
#07	Nonmalignant	4	33	2.80*E* + 07
#08	Malignant	10	22	6.00*E* + 07
#08	Nonmalignant	8	22	6.75*E* + 07
#09	Malignant	8	27	1.00*E* + 08
#09	Nonmalignant	8	26	1.00*E* + 07
#10	Malignant	6	28	3.80*E* + 07
#10	Nonmalignant	6	28	3.50*E* + 06
#11	Malignant	24	16	8.60*E* + 07
#11	Nonmalignant	12	16	5.60*E* + 06
#12	Malignant	6	29	4.50*E* + 06
#12	Nonmalignant	8	29	3.30*E* + 07
#13	Malignant	32	16	8.20*E* + 07
#13	Nonmalignant	n/a	n/a	n/a
#14	Malignant	12	12	9.50*E* + 07
#14	Nonmalignant	12	12	1.45*E* + 08
#15	Malignant	8	25	1.70*E* + 07
#15	Nonmalignant	8	25	1.00*E* + 07

n/a, nonmalignant tissue was not assessed.

## Data Availability

Access to the data for this study is restricted, and consideration will be given upon request.
